# Abnormal spontaneous neural activity in hippocampal–cortical system of patients with obsessive–compulsive disorder and its potential for diagnosis and prediction of early treatment response

**DOI:** 10.3389/fncel.2022.906534

**Published:** 2022-07-15

**Authors:** Haohao Yan, Xiaoxiao Shan, Huabing Li, Feng Liu, Wenbin Guo

**Affiliations:** ^1^Department of Psychiatry, National Clinical Research Center for Mental Disorders, The Second Xiangya Hospital of Central South University, Changsha, China; ^2^Department of Radiology, The Second Xiangya Hospital of Central South University, Changsha, China; ^3^Department of Radiology, Tianjin Medical University General Hospital, Tianjin, China; ^4^Department of Psychiatry, The Third People’s Hospital of Foshan, Foshan, China; ^5^Department of Psychiatry, Qiqihar Medical University, Qiqihar, China

**Keywords:** obsessive–compulsive disorder, regional homogeneity, hippocampus, parahippocampus, support vector machine

## Abstract

Early brain functional changes induced by pharmacotherapy in patients with obsessive–compulsive disorder (OCD) in relation to drugs *per se* or because of the impact of such drugs on the improvement of OCD remain unclear. Moreover, no neuroimaging biomarkers are available for diagnosis of OCD and prediction of early treatment response. We performed a longitudinal study involving 34 patients with OCD and 36 healthy controls (HCs). Patients with OCD received 5-week treatment with paroxetine (40 mg/d). Resting-state functional magnetic resonance imaging (fMRI), regional homogeneity (ReHo), support vector machine (SVM), and support vector regression (SVR) were applied to acquire and analyze the imaging data. Compared with HCs, patients with OCD had higher ReHo values in the right superior temporal gyrus and bilateral hippocampus/parahippocampus/fusiform gyrus/cerebellum at baseline. ReHo values in the left hippocampus and parahippocampus decreased significantly after treatment. The reduction rate (RR) of ReHo values was positively correlated with the RRs of the scores of Yale–Brown Obsessive–Compulsive Scale (Y-BOCS) and obsession. Abnormal ReHo values at baseline could serve as potential neuroimaging biomarkers for OCD diagnosis and prediction of early therapeutic response. This study highlighted the important role of the hippocampal–cortical system in the neuropsychological mechanism underlying OCD, pharmacological mechanism underlying OCD treatment, and the possibility of building models for diagnosis and prediction of early treatment response based on spontaneous activity in the hippocampal–cortical system.

## Introduction

Obsessive–compulsive disorder (OCD) is a severe and chronically disabling clinical condition with a lifetime prevalence rate of 2–3% (Sasson et al., 1997; Zohar, 1999; Ruscio et al., 2010). Increasing evidence suggests that OCD should be considered as a disorder of network connectivity rather than discrete cerebral regions (Hao et al., 2019). Cumulative and consistent evidence suggests that OCD is mediated by abnormal cortico-striato-thalamo-cortical (CSTC) circuits (Beucke et al., 2013). Several cerebral regions outside the CSTC circuits, such as the parietal cortex, insula, hippocampus, parahippocampus, and cerebellum, are also involved in the pathogenesis of OCD (Del Casale et al., 2011; Tang et al., 2015; Chen et al., 2016; Zhao et al., 2017; Yang et al., 2019), suggesting that the networks involved in OCD should be revisited. Recently, abnormal default mode network (DMN), frontal–parietal network, top–down control network, and salience network in OCD have attracted the attention of researchers (Göttlich et al., 2014; Gonçalves et al., 2016). However, outcomes of these studies were heterogeneous. The incomplete understanding of the neurophysiological mechanism underlying OCD has hampered efforts to enhance extant interventions and develop new treatments.

The pharmacological mechanism underlying the treatment for OCD remains unclear. The first-line treatments for OCD are selective serotonin reuptake inhibitors (SSRIs) and cognitive-behavioral therapy (CBT) (Bloch et al., 2010; Olatunji et al., 2013). Nevertheless, 30–40% of patients do not respond to SSRIs, and most patients do not generally achieve full remission (Fineberg et al., 2012, 2013). A recent article reviewed the neuroimaging studies about functional and structural neural changes in OCD after pharmacotherapy and found that drugs could generally affect the brains of patients with OCD (Atmaca, 2019). However, whether treatment-induced early neuroimaging changes are related to drugs *per se* or to the impact of such drugs on the improvement of OCD are unclear.

Thus far, no diagnostic biomarkers are available for OCD, and its diagnosis relies most on the recognition of characteristic symptoms assessed by clinical interview. Neuroimaging studies [such as resting-state functional magnetic resonance imaging (fMRI), task-based fMRI, positron emission tomography, and electroencephalogram] and electro-physiological studies (Riesel et al., 2011, 2015) have found various brain structural, functional, and metabolic abnormalities in patients with OCD compared with healthy controls (HCs). However, most studies have been conducted at the group level, thus limiting the diagnostic usage in clinical practice. In recent years, new statistical approaches, such as random forest models and multivariate pattern analysis (MVPA) techniques based on the individual level, have been developed, which may fill the research gap. A recent article reviewed the studies that applied MVPA techniques to neuroimaging data for distinguishing patients with OCD from HCs. Results showed that the accuracies ranged from 66 to 100% (Bruin et al., 2019) and indicated the enormous potential of MVPA techniques connected with neuroimaging studies in building models for the diagnosis of OCD.

Prediction of treatment response could optimize the use of medical resources, minimize exposure to ineffective drugs, and increase patient compliance. The prediction of treatment response for OCD has become an important research priority considering the prevalence and disability associated with it and the availability of different effective interventions. Although many studies have recognized demographic and psychometric characteristics correlated with treatment response in OCD, outcomes of predictions are inconsistent (Knopp et al., 2013; Alemany-Navarro et al., 2019). Neuroimaging features are promising for the prediction of treatment response for OCD (Salomoni et al., 2009; Sanematsu et al., 2010; Hoexter et al., 2013; Göttlich et al., 2015; Dunlop et al., 2016; Reggente et al., 2018). However, to the best of our knowledge, no studies have explored the possibility of using resting-state neuroimaging signals combined with machine learning techniques to predict early treatment response for OCD to paroxetine.

Thus, we designed the present longitudinal study, which compared the pre-treatment images with post-treatment images; for the latter, scanning was performed after 5-week treatment with paroxetine (40 mg/d). The 5-week treatment duration as the endpoint for observing early treatment response was appropriate for three reasons. First, a statistically significant benefit of SSRIs compared with placebo was observed within 2 weeks after the initial point of treatment in adults or children with OCD (Issari et al., 2016; Varigonda et al., 2016). Second, the practice guidelines for the treatment of OCD with serotonin reuptake inhibitors consider 8–12 weeks or at least 4–6 weeks at the highest comfortably tolerated dose as an adequate duration for a trial before switching drugs or augmenting therapy with another agent (American Psychiatric Association [APA], 2007). Third, to the best of our knowledge, no studies have measured early imaging changes (treatment duration < 12 weeks) after treatment with SSRIs in OCD. A recent systematic review involving studies performed from 1990 to 2020 to evaluate treatment-induced neuroimaging changes in brains of patients with OCD found that the treatment duration of all included studies varied from 12 weeks to 1 year (Bijanki et al., 2021). We aimed to explore neurophysiological mechanism underlying OCD; pharmacological mechanism of SSRIs on the treatment of OCD, especially for recognition of the pharmacotherapy-induced early functional brain changes that were related to drugs *per se* or to the impact of such drugs on the alleviation of OCD; and potential neuroimaging biomarkers for diagnosis and prediction of early treatment response.

We applied several techniques, namely, resting-state fMRI, regional homogeneity (ReHo), support vector machine (SVM), and support vector regression (SVR). Resting-state fMRI maps the spatiotemporal distribution of intrinsic spontaneous neural activity in a non-invasive and non-task-related manner. Since its discovery in 1992 (Ogawa et al., 1992), resting-state fMRI has rapidly become a vital tool in neuroscience research. ReHo was an extensively used and highly reliable approach to reveals the local functional connectivity (Zang et al., 2004). As a data-driven method and without any prior hypothesis, ReHo could access a high amount of information throughout the entire brain. SVM, one of the classical machine learning techniques (Meyer et al., 2003), was used to explore the possibility of abnormal ReHo values in patients with OCD to serve as neuroimaging biomarkers for diagnosis. SVM yields good performance in classification compared with classification trees, neural networks, or learning vector quantization (Meyer et al., 2003). SVR, a supervised machine learning technique, could efficiently resolve a non-linear regression problem by projecting the original feature into a kernel space, in which data were linearly discriminated (Ben-Hur et al., 2008). We used SVR to examine whether abnormal ReHo values at baseline could serve as potential neuroimaging biomarkers for the prediction of early treatment response.

This study hypothesized that abnormal ReHo values in the cerebral regions within the CSTC circuits and outside the CSTC circuits would be observed at baseline, and changes in ReHo values after 5 weeks of paroxetine treatment were correlated with the levels of the remission of OCD. We also hypothesized that abnormal ReHo values at baseline could serve as neuroimaging biomarkers for diagnosis and prediction of early treatment response.

## Materials and methods

### Participants

This study was approved by the Research Ethics Committee of the Second Xiangya Hospital of Central South University in China. The ethical code of the present study was 2018023. A written informed consent was acquired from each participant.

Thirty-six right-handed patients with drug-naive OCD were recruited from the Second Xiangya Hospital, and 36 right-handed HCs were recruited from the local community from January 2019 to September 2021. The diagnosis of OCD was confirmed by using the Structural Clinical Interview for Diagnostic and Statistical Manual of Mental Disorders-Fifth Edition (DSM-5) patient version (American Psychiatric Association [APA], 2013). HCs were screened using the Structured Clinical Interview for DSM-5 Axis I Disorders-Non-patient Edition. All participants were 18–49 years old and had more than 6 years of formal education. All patients with OCD had a Yale–Brown Obsessive–Compulsive Scale (Y-BOCS) score of more than 16 and a 17-item Hamilton Depression Rating Scale (HAMD-17) score of less than 18. All participants had no history of neurological illness or other major physical diseases, no history of any other psychiatric disorders, and no history of substance abuse disorders. Participants were excluded if they had significant medical conditions or were pregnant.

### Procedure

At baseline, all participants were scanned using a brain MRI scanner, and their general information was collected. Subsequently, patients with OCD received a paroxetine treatment for 5 weeks (40 mg/day) and were scanned again after the 5-week treatment. No other medicine and/or psychotherapy were involved during the 5-week period. Clinical symptoms of patients with OCD were assessed with Y-BOCS, Hamilton Depression Rating Scale (HAMD), Hamilton Anxiety Rating Scale (HAMA), Social Disability Screening Schedule (SDSS), Simplified Coping Style Questionnaire (CSQ), and Brief Cognitive Assessment Tool for Schizophrenia (B-CATS) at baseline and after treatment.

### Measures

Yale–Brown Obsessive–Compulsive Scale, a clinician-administered semi-structured interview, was scored on a five-point Likert scale (0–4, higher scores indicated greater disturbance). The total score was computed from the first 10 items (without items 1b and 6b). Items 1 to 5 measured obsession-related dysfunctions, whereas items 6 to 10 assessed compulsion-related dysfunctions. A validated Chinese version of Y-BOCS was applied in the present study (Zhang et al., 2019). The Chinese version of Y-BOCS had a high internal consistency as a whole (alpha = 0.90), as well as the obsessions (alpha = 0.87) and compulsions (alpha = 0.88) subscales. The test-retest reliability of the Chinese version of Y-BOCS was adequate (intraclass correlation coefficient = 0.63). Convergent validity of the Chinese version of Y-BOCS with the Clinical Global Impression-Severity (*r* = 0.75) and National Institute of Mental Health Global Obsessive Compulsive Scale was satisfactory (*r* = 0.71).

The 17-item HAMD (Bagby et al., 2004) was applied to assess depressive symptoms in patients with OCD, and higher scores indicated greater severity in depressive symptoms.

Hamilton Anxiety Rating Scale was used to measure the severity of anxiety symptoms in patients with OCD. HAMA included 14 items, which scored 0–4. A higher score indicated greater severity of anxiety symptoms.

Social Disability Screening Schedule was a 10-item clinician-administered scale to measure the degree of functional impairment. Each item was scored on a three-point Likert scale (0–2). Higher scores indicated greater functional impairment.

Simplified Coping Style Questionnaire (Xie, 1998) was based on the Ways of Coping questionnaire by Folkman and Lazarus (1988). It consisted of 20 items related to different approaches to cope with daily life events, and each item was scored on a four-point Likert scale (0–3). The CSQ contained two dimensions, namely, active coping (12 items) and passive coping (eight items).

Brief Cognitive Assessment Tool for Schizophrenia was an easily administered cognitive battery composed of four parts, namely, Digit Symbol Substitution Test, Trail-Making Test A, Trail-Making Test B, and Animal Fluency (Hurford et al., 2018). The Digit Symbol Substitution Test was scored with the number of correct number-symbol pairs. Trail-Making Test A and B were scored according to the time to completion. Animal Fluency was scored with the number of unique and appropriate answers.

### Imaging data acquisition and pre-processing

Functional MRI scans were obtained with a 3.0 T Philips scanner (Philips Achieva; Philips Medical Systems, Best; Netherlands) in the Second Xiangya Hospital. The participants were instructed to remain motionless and keep their eyes closed but remain awake. Soft earplugs and foam pads were used to reduce scanner noise and head motion. The scanning parameters were as follows: repetition time/echo time = 2000/30 ms, 33 slices, 64 × 64 matrix, 90° flip angle, 22 cm FOV, 4 mm-thick slice, no gap, and 240 volumes (8 min). The voxel size was 3.75 mm × 3.75 mm × 4 mm.

Data pre-processing was conducted in MATLAB^1^ with a data-processing assistant software (DPARSF) (Chao-Gan and Yu-Feng, 2010). The first 10 time points of each participant were removed for the participants to adapt to the scanning environment and signal equilibrium. The remaining 230 volumes underwent slice timing and head motion correction. All participants should not exceed the 2 mm maximum displacement in the x-, y-, or z-axis and the 2° angular motion in any direction to control the impact of head movement. Subsequently, the images were spatially normalized to the standard Montreal Neurological Institute (MNI) space by using the echo-planar imaging (EPI) template and resampled with a resolution of 3 × 3 × 3 mm^3^. The obtained images were bandpass filtered (0.01–0.08 Hz) and linearly detrended. A Band Pass Filter (BPF) was applied which could transmit all signals of frequencies within a given frequency band radians per second without any distortion and completely block all signals of frequencies outside this given frequency band. The frequency band (e.g., 0.01–0.08 Hz) was called the bandwidth of the BPF. The phase function of a BPF for the distortion less transmission was


$$\theta_{\left(\omega \right)}=-\omega \,t_{d}$$

where ω was the frequency, *t*_*d*_ was the time duration. The transfer function of a BPF was


$$\left|H_{\left(\omega \right)}\right|=\left\{ \begin{array}{c} 1\,f\,o\,r\,\left|\omega_{1}\right|<\omega <\left|\omega_{2}\right|\\ 0\,f\,o\,r\,\omega <\left|\omega_{1}\right|\,a\,n\,d\,\omega >\left|\omega_{2}\right| \end{array}\right.$$

The applied BPF filtered the data in the frequency domain by frequency sampling method, i.e., fast Fourier transform (FFT) transform to frequency domain, then selected the desired frequency band, and finally transformed back to time domain by inverse fast Fourier transform (iFFT).

### Regional homogeneity calculation

Regional homogeneity analysis was performed by using the DPARSF software. Individual ReHo maps were generated by calculating Kendall’s coefficient of concordance (KCC) to synchronize the time series of a given voxel with those of its 26 nearest neighbors. The formula for calculating the KCC value was described previously by Zang et al. (2004). The ReHo maps were normalized through the division of the KCC among each voxel by the average KCC of the whole brain for each subject to reduce the influence of individual variations. Subsequently, spatial smoothing was performed on the ReHo maps to improve the signal to noise ratio (SNR) (Mikl et al., 2008). Spatial smoothing was employed by convolving the fMRI signal with a Gaussian function of a specific width. The width was expressed in terms of Full Width at Half Maximum (FWHM). A 4 mm FWHM Gaussian kernel was applied as suggested in a previous study (Mikl et al., 2008).

### Statistical analysis

Differences in demographic data between patients with OCD and HCs were analyzed by using two-sample *t*-tests and a chi-square test as appropriate with the SPSS 19.0 software (SPSS Inc., Chicago, IL, United States). Paired *t*-tests were performed to compare the clinical symptoms of the patients at baseline with those after treatment. Normality of the difference of variable was assessed prior to using the paired *t*-tests. The Q-Q plots of the residual signal were provided (Supplementary Figure 1). Statistical significance was determined by *p* < 0.05. For the imaging data, statistical analyses were conducted with DPARSF. Two-sample *t*-tests were conducted on the individual normalized ReHo maps to distinguish clusters with abnormal ReHo values in patients with OCD in comparison with HCs. Each participant’s framewise displacement (FD) was calculated with the method in a previous study (Power et al., 2012). The mean FD, gender, age, and years of education were used as covariates of no interest in the two-sample *t*-tests. The ReHo values of these clusters between pre- and post-treatments in patients with OCD were compared using paired *t*-tests. The mean FD was used as covariates of no interest in the paired *t*-tests. In the two-sample and paired *t*-tests, the significance levels of the *p* values were corrected for multiple comparisons based on the Gaussian random field (GRF) theory (voxel significance: *p* < 0.001, cluster significance: *p* < 0.05) as suggested by a previous study (Chen et al., 2018).

### Correlation analysis

Pearson correlation analyses were performed to assess the relationship between abnormal ReHo values and the clinical data in patients with OCD at baseline. Before that, the normality of them was assessed (Supplementary Figure 2). The correlations between the percentage changes in clinical data (scores of Y-BOCS, obsessions, compulsions, HAMA, HAMD, SDSS, active and passive coping, Digit Symbol Substitution Test, Trail-Making Test A, Trail-Making Test B, and Animal Fluency) and the percentage changes in ReHo values of clusters were determined by Pearson correlation analyses.

### Support vector machine analysis

Classification analysis was performed to examine whether abnormal ReHo values at baseline could serve as imaging indicators to distinguish patients with OCD from HCs. Classification analysis was conducted using SVM in the LIBSVM software package (Chang and Lin, 2011). A Gaussian radial basis function kernel was applied. The C was the penalty parameter and γ was the free parameter in the radial basis function kernel. A “grid search” on C and γ was conducted using a cross-validation method. The pair of (C, γ) with the best cross-validation accuracy was picked (Liu et al., 2018). The cross-validation accuracy was the percentage of data which was correctly classified (Hsu et al., 2003). We performed permutation tests to assess whether the estimated accuracy differed from what was expected if the classifier was randomly assigning labels. By permuting the data labels and each shuffled instance used to train one SVM, we could estimate the distribution of the accuracy of classification under the null hypothesis (Gaonkar et al., 2015). In the permutation test, the probability of acquiring a given or more extreme of the accuracy of classification could be estimated by dividing the number of times, n*_*i*_*, the accuracy of classification obtained with the permuted data labels was higher or equal to the value of the accuracy of classification estimated with the true data labels, by the times of randomly permuted the data labels (n*_*p*_*). Thus, the equation of *p* values of statistical significance in the permutation test was $p=\max\left(\frac{1}{n_{p}},\frac{n_{i}}{n_{p}}\right)$ (Rosa et al., 2015). In the present study, n_*p*_ = 100,000.

### Support vector machine analysis

Support vector machine analysis was performed to determine whether abnormal ReHo values at baseline could predict early therapeutic response by using the LIBSVM software package (Chang and Lin, 2011). Actual treatment response to paroxetine was defined as reduction rate (RR) of Y-BOCS total scores, and subscale scores, including obsession and compulsion scores. The RR of Y-BOCS total scores was calculated according to the following formula: RR_Y–BOCS total_ = (Y-BOCS_total 0_ – Y-BOCS_total 5w_)/Y-BOCS_total 0_. RR_Y–BOCS total_ refers to the RR of Y-BOCS total scores after 5 weeks of treatment. Y-BOCS_total 0_ refers to the scores of Y-BOCS at baseline. Y-BOCS_total 5w_ refers to the scores of Y-BOCS after treatment. The RRs of obsession and compulsion scores were calculated in the same manner. Descriptions of algorithms and training set for SVR were presented in our previous study (Yan et al., 2022). In simple terms, we applied a leave-one-out cross-validation to select the components which maximized predictability of the model. A “grid search” method was used to access parameter optimization. The predicted treatment response was compared with the actual treatment response *via* the correlation coefficient. We also assessed the regression results with the Bland Altman plots.

## Results

### Demographic and clinical characteristics

Thirty-six patients and 36 HCs were enrolled in the present study. Two patients were excluded due to excessive head movement. Thirty-four patients and 36 HCs were included in the final analysis. Twenty-four of the 34 patients completed the 5-week follow-up. The main reason for dropping out was the inconvenience of participating in the follow-up during the COVID-19 pandemic. Detailed demographic and clinical data of the participants are shown in Table 1. No significant differences in age, gender, and years of education were observed between the OCD and control groups.

**TABLE 1:** Characteristics of participants.

Variables	Patients (*n* = 34) Mean and SD	Controls (*n* = 36) Mean and SD	*P*-value
Age (years)	26.94 ± 8.79	24.19 ± 4.32	0.11*^b^*
Sex (male/female)	20/14	20/16	0.78*^a^*
Years of education (years)	13.68 ± 2.79	14.50 ± 1.52	0.14*^b^*
Illness duration (months)	56.74 ± 67.56		
Y-BOCS	22.15 ± 5.13		
Obsessions	11.65 ± 3.21		
Compulsions	10.50 ± 3.38		
HAMD	11.71 ± 4.18		
HAMA	12.47 ± 5.41		
SDSS	5.15 ± 2.74		
**CSQ**			
Active coping	18.56 ± 7.37		
Passive coping	9.15 ± 4.43		
**B-CATS**			
Digit symbol substitution	54.85 ± 10.26		
Trail making test part A	35.15 ± 14.82		
Trail making test part B	67.97 ± 46.02		
Category fluency	17.82 ± 4.33		

*^a^The p-value for sex distribution was obtained by a chi-square test.*

*^b^The p-values were obtained by two samples t-tests.*

*SD, standard deviation; Y-BOCS, Yale–Brown Obsessive Compulsive Scale; HAMD, Hamilton Depression Rating Scale; HAMA, Hamilton Anxiety Rating Scale; SDSS, Social Disability Screening Schedule; CSQ, Simplified Coping Style Questionnaire; B-CATS, Brief Cognitive Assessment Tool for Schizophrenia.*

### Paroxetine treatment outcome

The clinical characteristics of 24 patients who completed the follow-up are shown in Table 2. The treatment duration of patients who completed the follow-up was 34 ± 5.23 days (from baseline to the second resting-state fMRI scan). After the 5-week treatment, patients with OCD showed significant clinical improvement relative to their baseline assessments. The scores of Y-BOCS (*p* < 0.001), subscales of Y-BOCS (*p* < 0.001), HAMD (*p* < 0.001), HAMA (*p* < 0.001), SDSS (*p* < 0.001), active coping (*p* = 0.019), and subscales of B-CATS, such as Digit symbol substitution (*p* = 0.002), Trail making test part A (*p* = 0.002), and Category fluency (*p* = 0.020), changed significantly. However, the scores of passive coping (*p* = 0.368) and Trail making test part B did not change significantly (*p* = 0.170).

**TABLE 2:** Clinical characteristics of patients with OCD who finished the follow-up.

Scales	Pre-treatment (*n* = 24) Mean and SD	Post-treatment (*n* = 24) Mean and SD	*P*
Y-BOCS	23.63 ± 4.34	17.13 ± 7.33	<0.001
Obsessions	12.54 ± 2.25	9.17 ± 3.42	<0.001
Compulsions	11.08 ± 3.11	7.96 ± 4.47	<0.001
HAMD	11.54 ± 4.18	5.92 ± 4.75	<0.001
HAMA	13.04 ± 4.72	7.04 ± 5.37	<0.001
SDSS	5.25 ± 2.29	2.63 ± 1.88	<0.001
**CSQ**			
Active coping	19.38 ± 7.84	21.67 ± 7.38	0.019
Passive coping	9.37 ± 4.73	9.92 ± 4.35	0.368
**B-CATS**			
Digit symbol substitution	55.50 ± 9.78	60.88 ± 10.78	0.002
Trail making test part A	34.38 ± 11.43	27.63 ± 8.68	0.002
Trail making test part B	63.13 ± 31.23	55.33 ± 22.05	0.170
Category fluency	18.08 ± 4.20	20.46 ± 3.19	0.020

*OCD, obsessive–compulsive disorder; SD, standard deviation; Y-BOCS, Yale–Brown Obsessive Compulsive Scale; HAMD, Hamilton Depression Rating Scale; HAMA, Hamilton Anxiety Rating Scale; SDSS, Social Disability Screening Schedule; CSQ, Simplified Coping Style Questionnaire; B-CATS, Brief Cognitive Assessment Tool for Schizophrenia.*

### Regional homogeneity analysis in pre-treatment patients and healthy controls

**FIGURE 1:**
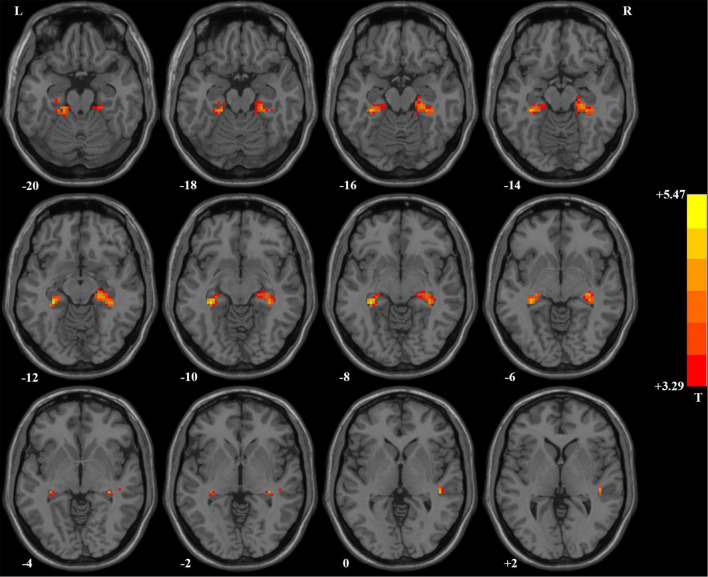
Brain regions with significant difference in ReHo values between patients with OCD and healthy controls. Compared with healthy controls, patients with OCD had higher ReHo values in the right superior temporal gyrus and bilateral hippocampus/parahippocampus/fusiform gyrus/cerebellum. ReHo, regional homogeneity; OCD, obsessive–compulsive disorder.

Compared with HCs, patients with OCD had higher ReHo values in the right hippocampus/parahippocampus/fusiform gyrus/cerebellum, left hippocampus/parahippocampus/fusi form gyrus/cerebellum, and right superior temporal gyrus (STG) at baseline. Detailed information is presented in Table 3 and Figure 1. No significant difference in mean FD was found between patients and HCs. The signal of the blood-oxygen-level dependent (BOLD) suffers the magnetic susceptibility artifacts, particularly in the STG. These artifacts appear as geometric distortions and signal loss in the T2*-weighted EPI. To assess the detectability of fMRI signals, the temporal SNR (tSNR) of the three clusters with abnormal ReHo values was calculated. The tSNR served as a quantitative indicator of the temporal characteristics of fMRI time-course data which could be used to measure the detectability of fMRI signals (Murphy et al., 2007). The tSNR of a time series x*_*i*_* was defined by:


$$\text{tSNR}=\frac{\mu }{\sigma }=\frac{\mu }{\sqrt{1/N\,\sum_{i=1}^{N}{\left(x_{i}-\mu \right)}^{2}}}$$

**TABLE 3:** Regions with abnormal ReHo values in patients with OCD at baseline and alterations of ReHo values after treatment.

Cluster location	Peak (MNI)			Number of voxels	*T* value
	
	x	y	z		
**Patients with OCD at baseline versus controls**					
Right Hippocampus/Parahippocampus/Fusiform Gyrus/Cerebellum	24	−27	−15	114	4.7262
Left Hippocampus/Parahippocampus/Fusiform Gyrus/Cerebellum	−33	−33	−9	104	5.4679
Right Superior Temporal Gyrus	45	−30	0	26	4.9702
**OCD patients after 5-week treatment versus at baseline**					
Left Hippocampus/Parahippocampus	−24	−24	−9	37	−1.97837

*OCD, obsessive–compulsive disorder; MNI, Montreal Neurological Institute; ReHo, regional homogeneity.*

where μ was the mean of the time series, δ was its standard deviation, and N was the number of time points. In the present study, the number of time points after pre-processing of imaging data was 230. We repeated the two-sample *t*-tests to assess the impact of possible artifacts on results, in which the mean FD, gender, age, years of education, and tSNR were used as covariates of no interest. The results did not change. The higher ReHo values in the right STG after adding the tSNR as covariate in the two-sample *t*-test were shown in Supplementary Figure 3.

### Support vector machine analysis result

**FIGURE 2:**
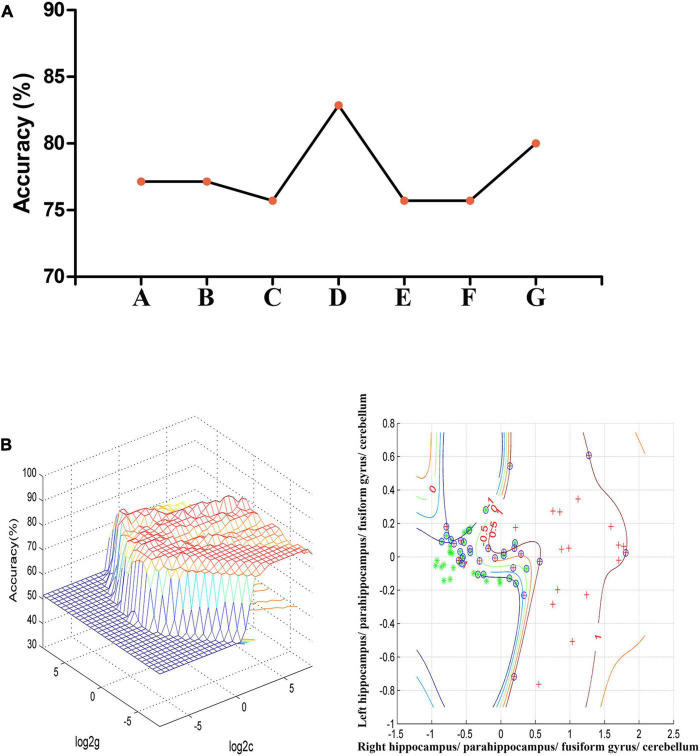
**(A)** The accuracy of classification of seven combinations of ReHo values. A represents the right hippocampus/parahippocampus/fusiform gyrus/cerebellum; B represents the left hippocampus/parahippocampus/fusiform gyrus/cerebellum; C represents the right superior temporal gyrus; D represents the right hippocampus/parahippocampus/fusiform gyrus/cerebellum and left hippocampus/parahippocampus/fusiform gyrus/cerebellum; E represents the right hippocampus/parahippocampus/fusiform gyrus/cerebellum and right superior temporal gyrus; F represents the left hippocampus/parahippocampus/fusiform gyrus/cerebellum and right superior temporal gyrus; G represents the right hippocampus/parahippocampus/fusiform gyrus/cerebellum, left hippocampus/parahippocampus/fusiform gyrus/cerebellum, and right superior temporal gyrus. **(B)** SVM analysis of the combination of ReHo values in the right hippocampus/parahippocampus/fusiform gyrus/cerebellum and left hippocampus/parahippocampus/fusiform gyrus/cerebellum. Sensitivity = 73.53%, specificity = 91.67%, and accuracy = 82.86%. SVM, support vector machines; ReHo, regional homogeneity.

In the combination of ReHo values in the right hippo campus/parahippocampus/fusiform gyrus/cerebellum and left hippocampus/parahippocampus/fusiform gyrus/cerebellum, 58 out of 70 participants were accurately classified with the highest accuracy. This combination was the optimal combination with a sensitivity of 73.53%, a specificity of 91.67%, and an accuracy of 82.86% [Figure 2; confidence interval (CI) 95% = 73.44–92.29%; *p* < 0.001, a permutation test with 100,000 repetitions]. All the combinations had a sensitivity = 67.64 ± 6.35%, a specificity = 87.30 ± 4.50%, and an accuracy = 77.75 ± 2.72% (Figure 2).

### Regional homogeneity analysis in pre-treatment and post-treatment patients with obsessive–compulsive disorder

**FIGURE 3:**
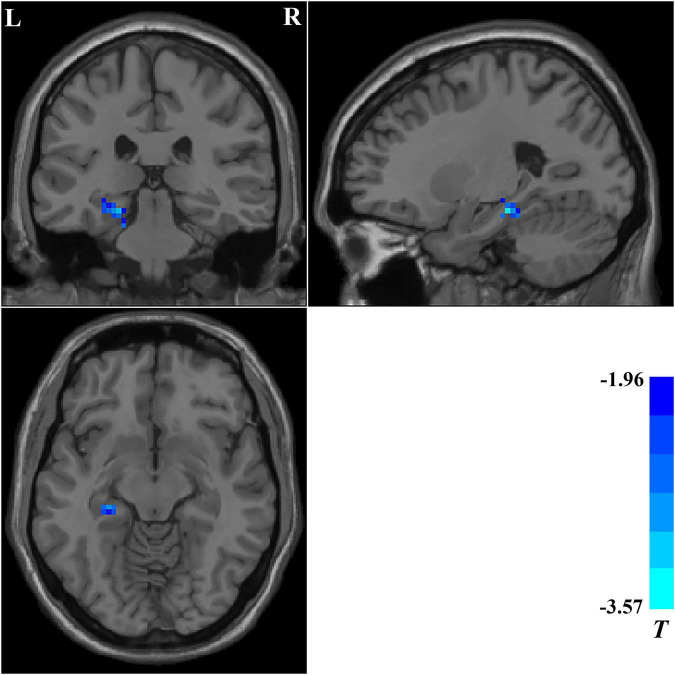
Brain regions showing significant changes in ReHo values after treatment. Compared with the baseline data, patients with OCD showed significantly decreased ReHo values in the left hippocampus and parahippocampus after a 5-week paroxetine treatment. ReHo, regional homogeneity; OCD, obsessive–compulsive disorder.

We compared the ReHo values of patients with OCD who completed follow-up in brain regions with abnormal ReHo values at baseline. Compared with baseline data, patients with OCD showed significantly decreased ReHo values after 5-week paroxetine treatment in the left hippocampus and parahippocampus (Table 3 and Figure 3).

### Support vector regression analysis result

**FIGURE 4:**
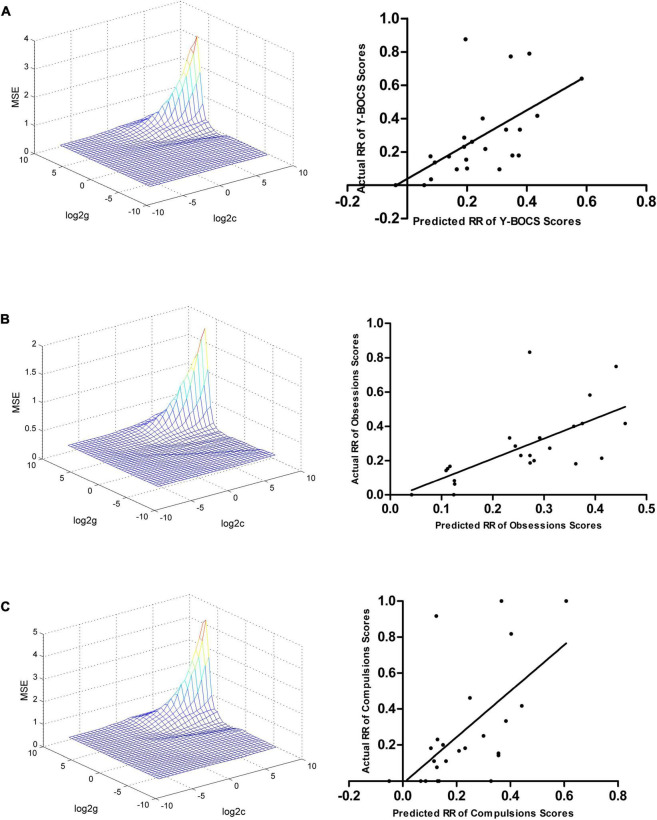
Support vector regression results suggested that the ReHo values in the left hippocampus, parahippocampus, fusiform gyrus, and cerebellum at baseline can be used to predict early therapeutic response. **(A)** SVR results showed a positive correlation between the actual and predicted RRs in the total scores of Y-BOCS (*r* = 0.590, *p* = 0.002). **(B)** SVR results showed a positive correlation between the actual and predicted RRs in scores of obsession (*r* = 0.661, *p* < 0.001). **(C)** SVR results showed a positive correlation between the actual and predicted RRs in the scores of compulsion (*r* = 0.595, *p* = 0.002). SVR, support vector regression; ReHo, regional homogeneity; RR, reduction rate.

The ReHo values in the left hippocampus/parahippocampus/fusiform gyrus/cerebellum at baseline could be used to predict early therapeutic response. The SVR results showed positive correlations between actual and predicted RRs in the total scores of Y-BOCS (*r* = 0.590, *p* = 0.002, Figure 4A), and scores of obsession (*r* = 0.661, *p* < 0.001, Figure 4B) and compulsion (*r* = 0.595, *p* = 0.002, Figure 4C). The significance threshold was *p* = 0.017 after Bonferroni correction (0.05/3 for the three types of Y-BOCS scores). The Bland Altman plots of the regression results were shown in the Supplementary Figure 4.

### Correlation between regional homogeneity values and clinical symptoms of patients with obsessive–compulsive disorder

No significant correlation was found between abnormal ReHo values and clinical symptoms (illness duration, the scores of Y-BOCS, obsessions, compulsions, HAMD, HAMA, SDSS, subscales of CSQ, and subscales of B-CATS) in patients with OCD at baseline.

The RR of the ReHo values in the left hippocampus and parahippocampus, where ReHo values decreased significantly after the 5-week paroxetine treatment, was positively correlated with the RRs of the scores of Y-BOCS (*r* = 0.407, *p* = 0.049.) and obsession (*r* = 0.422, *p* = 0.040).

## Discussion

This longitudinal study found that drug-naive patients with OCD had higher ReHo values in the right STG, and bilateral hippocampus/parahippocampus/fusiform gyrus/cerebellum than HCs. After the 5-week treatment with paroxetine (40 mg/day), patients showed significant clinical improvement in the symptoms of OCD, depressive symptoms, anxiety symptoms, social function, active coping, and cognition compared with their baseline assessment. The elevated ReHo values in the left hippocampus and parahippocampus decreased significantly after treatment, and the RR of ReHo values was positively correlated with the RRs of the scores of Y-BOCS and obsession. The SVR results showed that the ReHo values in the left hippocampus/parahippocampus/fusiform gyrus/cerebellum at baseline could be applied to predict early therapeutic response. The ReHo values in the right hippocampus/parahippocampus/fusiform gyrus/cerebellum and left hippocampus/parahippocampus/fusiform gyrus/cerebellum at baseline could be used to distinguish patients with OCD from HCs with high accuracy, sensitivity, and specificity.

Cumulative evidence suggested that abnormal CSTC circuits were not the only important pathological basis of OCD. Other cerebral regions outside the CSTC circuits were involved in OCD, such as the parietal cortex, insula, cerebellum, hippocampus, parahippocampus, fusiform gyrus, and STG (Del Casale et al., 2011; Zhao et al., 2017; Hao et al., 2019; Xia et al., 2019; Yang et al., 2019). A previous study suggested that a combination of amplitude of low-frequency fluctuation (ALFF) and ReHo which depicted different regional characteristics of resting-state fMRI might yield a more comprehensive pathophysiological framework for mental disorders (Sroubek et al., 2013). Thus, we measured ALFF in the present study. Compared with HCs, patients with OCD had higher ALFF values in the CSTC circuits such as the orbitofrontal cortex, superior medial frontal cortex, and anterior cingulate cortex (Supplementary Figure 5 and Supplementary Table 1). However, abnormal ALFF values in the patients with OCD did not change significantly after treatment (Supplementary Table 1).

### Increased regional homogeneity values in the right superior temporal gyrus in patients with obsessive–compulsive disorder at baseline

Increased brain spontaneous activities in the right STG in patients with OCD were found at baseline. Evidence suggested the existence of hemispheric bias in STG, with a preference for the right STG in processing social and emotional stimuli and for the left STG in processing language stimuli (Narumoto et al., 2001; Redcay, 2008). Xia et al. (2019) reported that OCD patients with anhedonia showed significantly decreased ALFF in the right STG compared with OCD patients without anhedonia, and the ALFF values in the right STG were negatively correlated with the severity of social anhedonia. Anatomically, the STG is directly connected with the caudate nucleus, which is one of the central nodes for reward processing (Yeterian and Pandya, 1998). Abnormal functional connectivity between the caudate nucleus and the right STG in patients with OCD has been reported (Chen et al., 2016). McLellan et al. (2018) reported that adolescents with treatment-resistant depression and history of suicide attempt had reduced right STG volume compared with HCs. Pan et al. (2015) also found that patients with major depressive disorder and a history of suicide attempt had lower right STG volume compared with HCs. These findings suggested that the right STG was an important region for emotional regulation. Park et al. (2011) found that the right STG served as a central region for the network nodes responsible for retaining stimulus through gamma activity, suggesting that the right STG had an important role in working memory. Paulus et al. (2005) found that bilateral STG showed increased activity in a decision-making situation, suggesting that STG was involved in information assessment and action selection. In summary, increased brain spontaneous activities in the right STG might be a compensatory mechanism for impaired emotional regulation, working memory, and decision-making in patients with OCD (Abramovitch et al., 2013; Robbins et al., 2019).

### Increased regional homogeneity values in the bilateral fusiform gyrus in patients with obsessive–compulsive disorder at baseline

We found increased brain spontaneous activities in the bilateral fusiform gyrus in patients at baseline. Previous studies reported that patients with OCD had abnormal local spontaneous activities (Yang et al., 2010; Hao et al., 2019) and functional connectivity (Ciesielski et al., 2012; Beucke et al., 2014) in the fusiform gyrus. The fusiform gyrus is commonly considered a key structure for high-level vision, such as face perception, reading, and object recognition (Kanwisher et al., 1997; McCandliss et al., 2003; Weiner and Zilles, 2016). Evidence suggested that fusiform gyrus had an important role in visuospatial and visuoperceptual working memory (Druzgal and D’Esposito, 2001; Sala-Llonch et al., 2015), social perception (Schultz, 2005), social cognition (Schultz et al., 2003), and emotional response inhibition (Berlin et al., 2015). OCD might be a consequence of dysfunctional circuits for response inhibition, working memory, cognitive flexibility, error monitoring, and planning (goal-directed behavior) (Abramovitch et al., 2013; Robbins et al., 2019). Our findings implied that the fusiform gyrus was a crucial structure in the neuropsychological mechanism underlying OCD.

### Increased regional homogeneity values in the bilateral cerebellum in patients with obsessive–compulsive disorder at baseline

Consistent with previous studies (Ping et al., 2013; Yang et al., 2015; Chen et al., 2016), increased ReHo values in the bilateral cerebellum were found in patients with OCD at baseline. Previous studies revealed the important role of cerebellum in attention (Schweizer et al., 2007; Mannarelli et al., 2019). Increased brain spontaneous activities in the cerebellum might be a compensatory mechanism for impaired attention, especially selective attention, which was identified to be central to the symptomatology of OCD (Clayton et al., 1999; Muller and Roberts, 2005). Previous studies also suggested the function of emotional regulation in the cerebellum (Schutter and van Honk, 2009; Baumann and Mattingley, 2012). The dysfunction of emotional regulation might contribute to the maintenance of distress and anxiety in patients with OCD (Fergus and Bardeen, 2014; Stern et al., 2014). Cerebellum has a crucial role in fear conditioning (Sacchetti et al., 2002, 2005), prediction, especially prediction errors (Schlerf et al., 2012; Ernst et al., 2019), response inhibition (Mostofsky et al., 2003; Wynn et al., 2019), and working memory (Durisko and Fiez, 2010; Ashida et al., 2019). Increased brain spontaneous activities in the cerebellum might be a compensatory mechanism for impaired fear conditioning, response inhibition, and working memory and could be used to explain the obsessive thoughts and repetitive behaviors that originated from excessive error prediction.

### Elevated regional homogeneity values in the bilateral hippocampus and parahippocampus decreased significantly after treatment

A previous longitudinal study found that patients with OCD had higher ALFF values in the left hippocampus/parahippocampus than HCs at baseline, and the differences disappeared after a 4-week CBT treatment (Zhao et al., 2017). We found increased ReHo values in the bilateral hippocampus and parahippocampus in patients at baseline. Moreover, after the 5-week treatment with paroxetine, the elevated ReHo values in the left hippocampus and parahippocampus decreased significantly. The hippocampal–cortical system, including the hippocampal region and its widespread cortical targets, might provide the neural building blocks for simulating upcoming events during planning and decision-making (prediction) and when imagining novel scenarios (imagination) (Buckner, 2010; Stachenfeld et al., 2017). In the hippocampal–cortical system, the hippocampal region was connected to the cortical regions through the adjacent entorhinal cortex, which was projected to the parahippocampal and perirhinal cortices. As a result, the parahippocampal and perirhinal cortices were projected to widespread cortical areas. Increased brain spontaneous activities in the hippocampus/parahippocampus might partly explain the excessive worries and anxiety over upcoming events through inappropriate imagination and prediction in OCD (O’Connor and Aardema, 2003). There was other evidence about the increased brain spontaneous activities in the parahippocampus mediated the excessive worries and anxiety over upcoming events in OCD. A task-based fMRI study was performed to test which brain regions contributed to suspicion in a repeated bargaining game and found that activations in the bilateral parahippocampus were associated with suspicion. Furthermore, less credibility appeared when more sensitive parahippocampal activation was observed (Bhatt et al., 2012). Thus, the parahippocampus might contribute to suspicion; excessive suspicion might be the basis for obsessive thoughts and repetitive behaviors and the accompanying excessive worries and anxiety for upcoming events in OCD. In addition, the hippocampus played a crucial role in response inhibition (Davidson and Jarrard, 2004) and working memory (Leszczynski, 2011; Yonelinas, 2013). Task-based fMRI (Luck et al., 2010) and lesion studies (Hasselmo and Stern, 2006; Smits et al., 2009) suggested that the parahippocampus had a critical role in working memory. Increased brain spontaneous activities in the hippocampus/parahippocampus might serve as compensatory mechanisms for impaired response inhibition and working memory. The hippocampus plays an important role in contextual fear conditioning (Anagnostaras et al., 2001; Sanders et al., 2003). Dysfunction of fear acquisition and extinction is a core perspective in the cognitive behavioral model of OCD, which underlies exposure-based CBT (McGuire et al., 2016; Cooper and Dunsmoor, 2021). In a task-based fMRI study performed by Shapira et al., 2003, patients with OCD showed significantly increased activation in the left parahippocampus compared with HCs when subjected to disgust-inducing stimuli. This finding suggested that the parahippocampus contributed to the arousal of disgust, which was an important symptomatology of OCD (Knowles et al., 2018). In summary, our findings highlighted the crucial role of the hippocampal–cortical system in the neuropsychological mechanism of OCD.

Elevated ReHo values in the left hippocampus/parahippocampus decreased significantly after the 5-week treatment with paroxetine, and the RR of the ReHo values in the left hippocampus/parahippocampus was positively correlated with the RRs of the scores of Y-BOCS and obsession. These findings implied the important role of the hippocampus and parahippocampus in the pharmacological mechanism underlying SSRIs in the treatment of OCD. Early brain functional changes induced by pharmacotherapy (normalization in the present study) were related to the effects of drugs on the improvement of OCD (treatment effects) rather than their side effects. Consistent with our findings, Gilbert et al. (2000) reported that the greater thalamic volume in patients with OCD than HCs was reduced significantly after 12-week treatment with paroxetine and the reduction of the thalamic volume was associated with the decrease in symptomatic severity. Rosenberg et al. (2000) found that the higher glutamatergic concentration in the caudate of pediatric patients with OCD than HCs decreased significantly after 12-week treatment with paroxetine. Denormalization in brains of patients with OCD after treatment with SSRIs was also observed in the previous studies. Hansen et al. (2002) reported that regional cerebral glucose metabolic rate in the right caudate nucleus of patients with OCD reduced significantly after 12–20 weeks of treatment with paroxetine. Lázaro et al. (2008) observed that patients with OCD had decreased activation in the left putamen and insula in response to the serial reaction task after 6-month treatment with fluoxetine (activation in the left putamen and insula in response to the serial reaction task in patients with OCD before treatment showed no significant difference related to HCs). These normalization and denormalization in brains of patients with OCD could be explained by treatment effects and side effects of pharmacotherapy, respectively (Abbott et al., 2013).

Furthermore, the ReHo values in the left hippocampus/parahippocampus/fusiform gyrus/cerebellum at baseline can be applied to the prediction of early therapeutic response. Sanematsu et al. (2010) found that during symptom provocation task, patients with OCD showed brain activation in the right cerebellum, and the pre-treatment activation in the right cerebellum could be used to predict the improvement in the Y-BOCS score after the 12-week treatment with fluvoxamine. Although different SSRIs were used in the treatments of OCD, their results were consistent with our findings.

### Strengths and limitations

This study was the first to apply resting-state fMRI technique combined with local spontaneous brain activity analysis (ReHo analysis) and methods of machine learning, including SVM and SVR analyses, to explore the neuropsychological mechanism underlying OCD, pharmacological mechanism underlying OCD treatment, and the possibility of building models for the diagnosis and prediction of early treatment response. Drug-naïve patients were recruited, and the age range was from 18 to 49 years old to avoid the impacts of drug, neurodevelopment, and aging on local spontaneous brain activity. However, several limitations were noted. First, this monocentric study with a relatively small sample size and high drop-out rate due to the inconvenience of participation in the follow-up during the COVID-19 pandemic might restrict the possibility of the generalization of these outcomes to other centers. Second, we only performed a 5-week follow-up for the patients, who were treated with paroxetine. Although the patients showed a significant improvement in clinical symptoms after treatment, the insufficient treatment might result in a false negative outcome in the normalization of increased ReHo values. Third, although the tSNR was measured which could be used to assess the detectability of fMRI signals, the possible artifacts could bias the results. Fourth, although the cerebellum was involved in the neurophysiological mechanism underlying OCD, with a slice thickness of 4 mm it might be difficult to infer precise localization within the cerebellum. Fifth, denormalization in brains of patients with OCD related to side effects of SSRIs was reported previously which was opposite to our findings. Besides, other domains of side effects of the SSRIs use were reported before. Sayyah et al. (2016) reported that SSRIs use could cause cognitive dysfunction in patients with OCD or depression in the acute phase of treatment. Skandali et al. (2018) reported that acute escitalopram administration on healthy volunteers impaired functions of cognitive flexibility and learning. Thus, explaining normalization of ReHo values in the left hippocampus/parahippocampus of patients with OCD after 5-week treatment with paroxetine should be cautious. Sixth, previous studies reported numerous abnormalities of distant functional connectivity in patients with OCD (Kaufmann et al., 2013; Beucke et al., 2014). We cannot measure abnormal distant functional connectivity using the ReHo indicator in the present study.

## Conclusion

This study highlighted the important role of the hippocampal–cortical system in the neuropsychological mechanisms underlying OCD and the pharmacological mechanism underlying the treatments of OCD, as well as the possibility of building models for the diagnosis and prediction of early treatment response based on the spontaneous activity in the hippocampal–cortical system. This study also revealed that the early functional brain changes induced by pharmacotherapy might be related to the treatment effects rather than the drugs *per se* or their side effects.

## Data Availability Statement

The raw data supporting the conclusions of this article will be made available by the authors, without undue reservation.

## Ethics statement

The studies involving human participants were reviewed and approved by Research Ethics Committee of Second Xiangya Hospital of Central South University in China. The patients/participants provided their written informed consent to participate in this study.

## Author contributions

HY: methodology, data curation, formal analysis, and writing – review and editing. XS: conceptualization, data curation, and writing – review and editing. HL: data curation. FL: conceptualization and funding acquisition. WG: methodology, data curation, writing – review and editing, and funding acquisition. All authors contributed to the article and approved the submitted version.

## Conflict of Interest

The authors declare that the research was conducted in the absence of any commercial or financial relationships that could be construed as a potential conflict of interest.

## Publisher’s Note

All claims expressed in this article are solely those of the authors and do not necessarily represent those of their affiliated organizations, or those of the publisher, the editors and the reviewers. Any product that may be evaluated in this article, or claim that may be made by its manufacturer, is not guaranteed or endorsed by the publisher.

## Footnotes

1
http://www.mathworks.com
