# Supplementary material for: Abnormal spontaneous neural activity in hippocampal–cortical system of patients with obsessive–compulsive disorder and its potential for diagnosis and prediction of early treatment response
Source: Front Cell Neurosci. 2022 Jul 15;16:906534. doi: 10.3389/fncel.2022.906534 (PMC9334680; doi:10.3389/fncel.2022.906534)
Supplement: Supplementary file 1 [file Table_1.docx]

Table S1. Regions with abnormal ALFF values in patients with OCD at baseline and alterations of ALFF values after treatment.

| Cluster location | Peak (MNI) | | | Number of voxels | *T* value |
| --- | --- | --- | --- | --- | --- |
|  | x | y | z |  |  |
| Patients with OCD at baseline versus controls | | | | | |
| Right Insula/ Orbitofrontal Cortex | 39 | 27 | 0 | 38 | 4.0885 |
| Right ACC/MCC/SFG | 9 | 36 | 33 | 33 | 3.9717 |
| Left Insula | -33 | 24 | 9 | 28 | 3.8528 |
| Left SMFC | -6 | 57 | 18 | 26 | 4.0524 |
| OCD patients after 5-week treatment versus at baseline | | | | | |
| None |  |  |  |  |  |

OCD = obsessive–compulsive disorder; MNI = Montreal Neurological Institute; ALFF = Amplitude of low-frequency fluctuation; ACC = anterior cingulate cortex; MCC = middle cingulate cortex; SFG = superior frontal gyrus; SMFC = superior medial frontal cortex.

The significance level was set at *p* < 0.05 for multiple comparisons corrected by Gaussian Random Field (GRF) theory (voxel significance: *p* < 0.001, cluster significance: *p* < 0.05). Age, education level and mean framewise displacement (FD) were used as covariates to minimize the potential effects of these variables
