# Supplementary figures and images for: Abnormal spontaneous neural activity in hippocampal–cortical system of patients with obsessive–compulsive disorder and its potential for diagnosis and prediction of early treatment response
Source: Front Cell Neurosci. 2022 Jul 15;16:906534. doi: 10.3389/fncel.2022.906534 (PMC9334680; doi:10.3389/fncel.2022.906534)

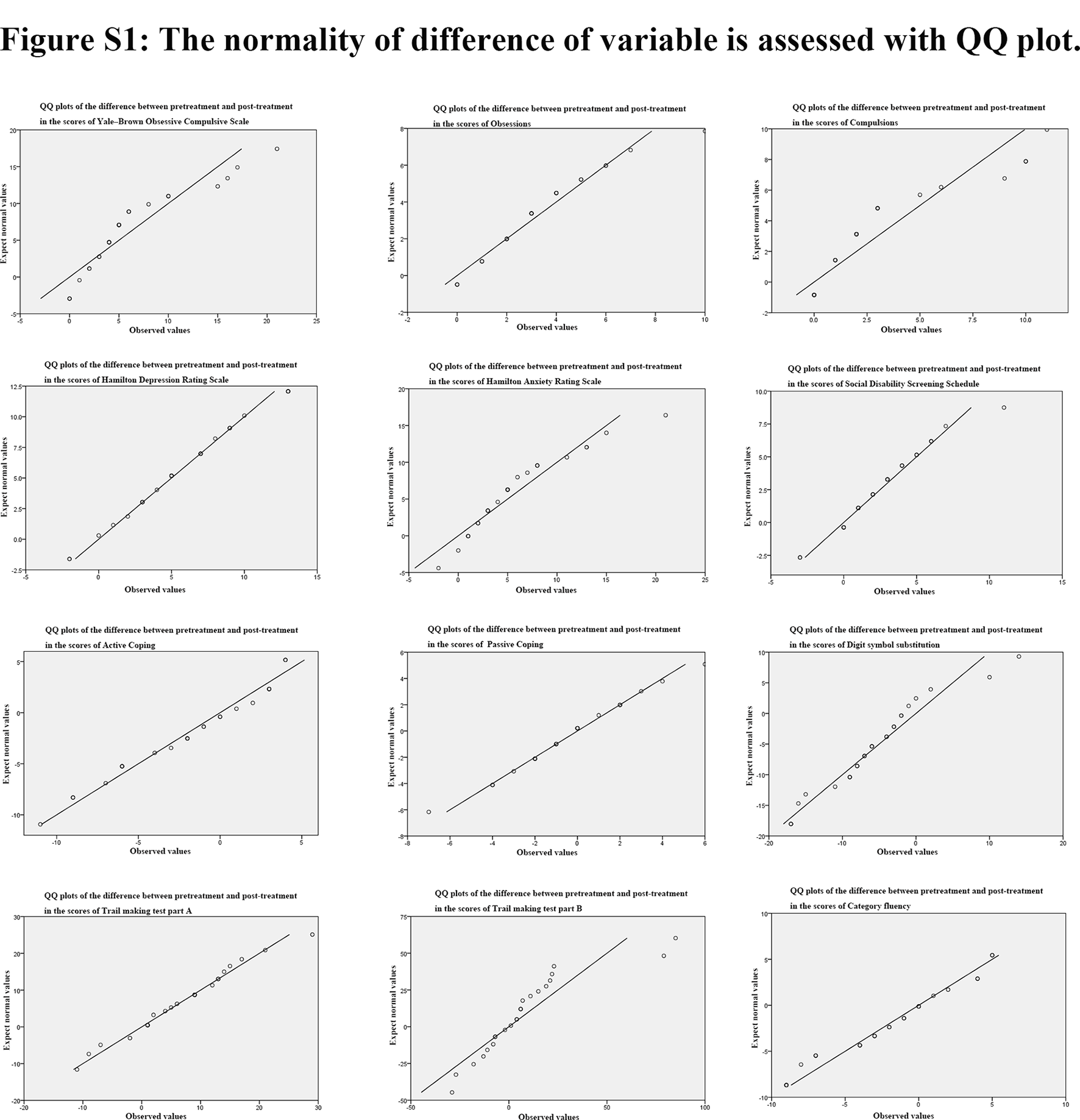

Supplement: Supplementary file 2 [file Image_1.TIF]

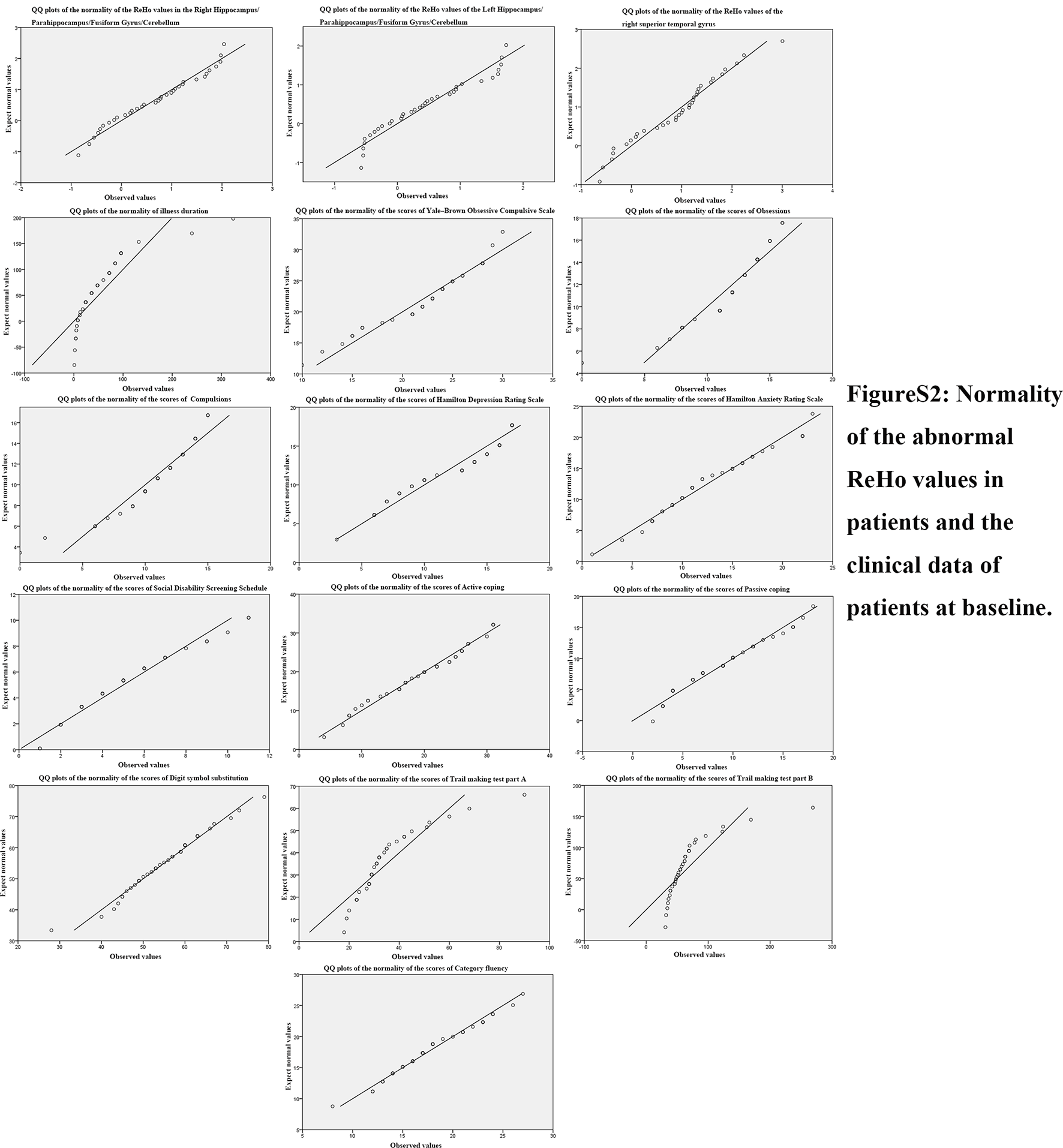

Supplement: Supplementary file 3 [file Image_2.TIF]

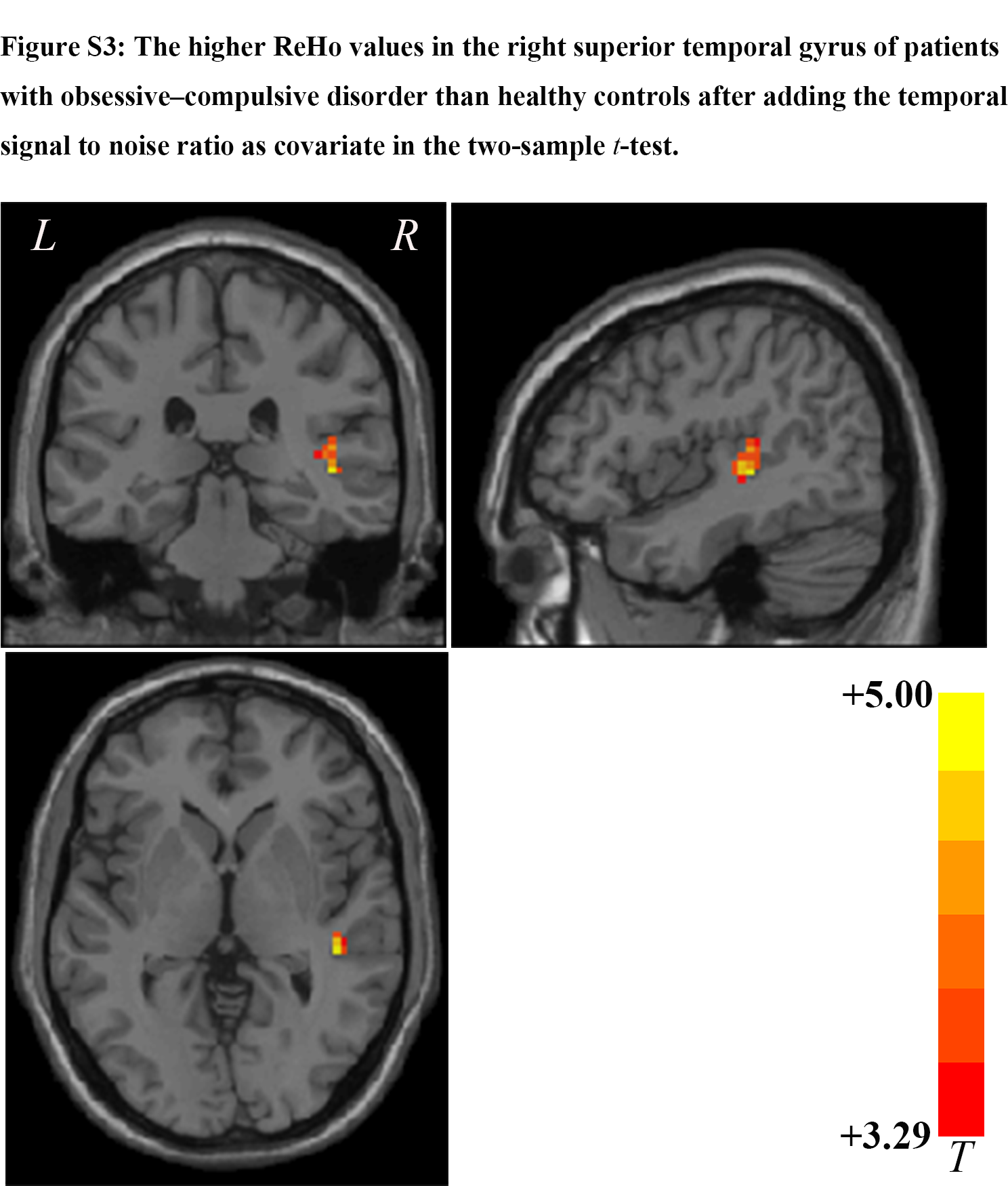

Supplement: Supplementary file 4 [file Image_3.TIF]

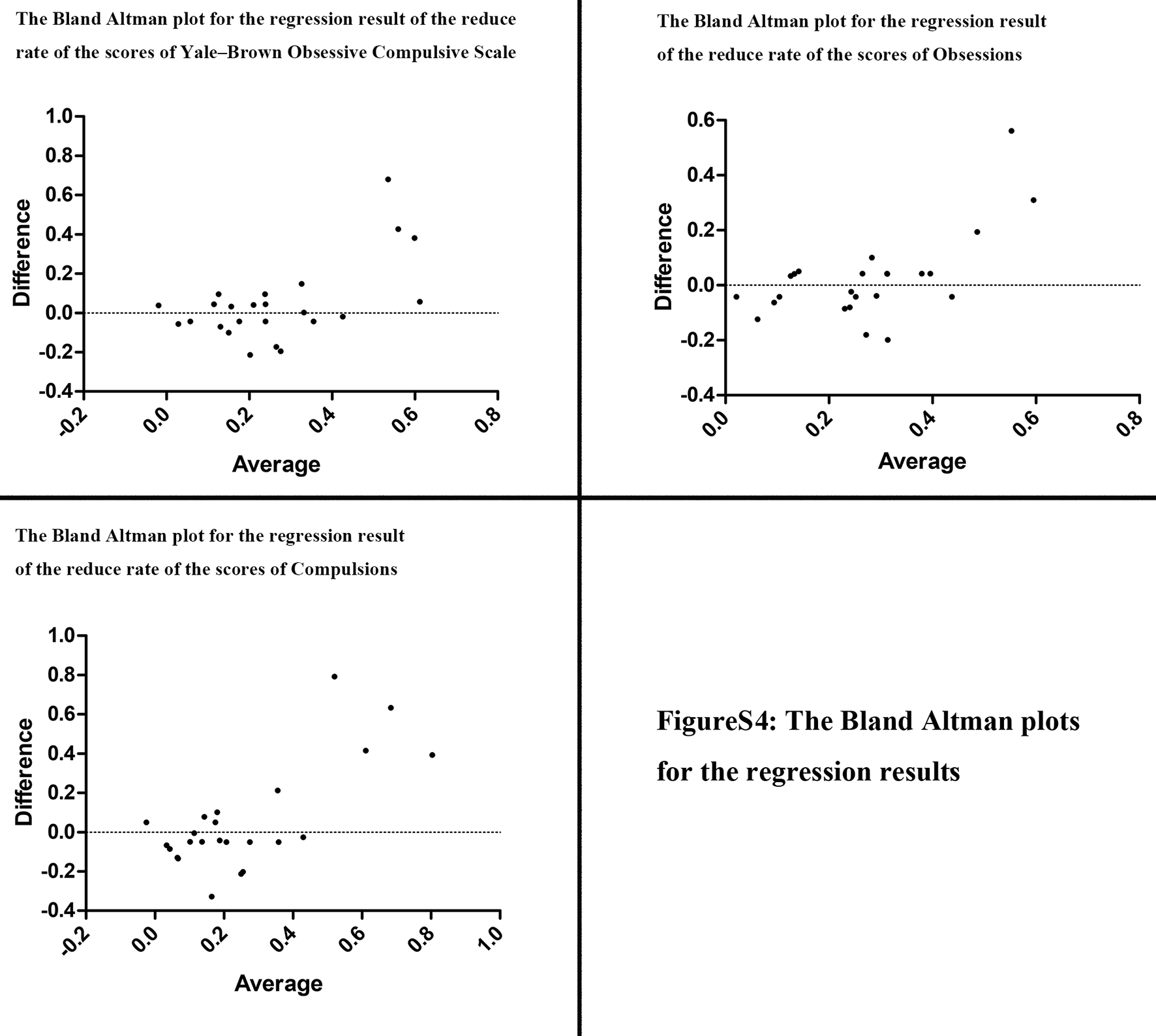

Supplement: Supplementary file 5 [file Image_4.TIF]

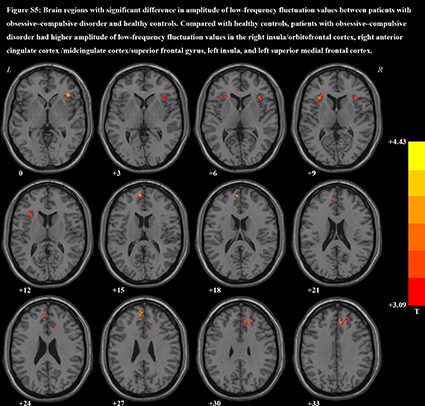

Supplement: Supplementary file 6 [file Image_5.TIF]
